# Supplementary material for: HIVprotI: an integrated web based platform for prediction and design of HIV proteins inhibitors
Source: J Cheminform. 2018 Mar 9;10:12. doi: 10.1186/s13321-018-0266-y (PMC5845081; doi:10.1186/s13321-018-0266-y)
Supplement: Supplementary file 2 — Additional file 2. Source code of HIVProtI web server. [file 13321_2018_266_MOESM2_ESM.zip › HIVprotI_Source-code/hivprotI/weka-3-6-6/doc/weka/filters/unsupervised/instance/subsetbyexpression/Parser.html]

Parser


---


|  |  |  |
| --- | --- | --- |
| **Overview** \| **Package** \| **Class** \| **Tree** \| **Deprecated** \| **Index** \| **Help** \| **Weka's home** | |  |
| PREV CLASS   **NEXT CLASS** | **FRAMES**    **NO FRAMES**     **All Classes** |  |
| SUMMARY: NESTED \| FIELD \| CONSTR \| METHOD | DETAIL: FIELD \| CONSTR \| METHOD |  |


---


## weka.filters.unsupervised.instance.subsetbyexpression Class Parser

```
java.lang.Object
  java_cup.runtime.lr_parser
      weka.filters.unsupervised.instance.subsetbyexpression.Parser
```

---

``` public class Parser extends java_cup.runtime.lr_parser ```

CUP v0.11a beta 20060608 generated parser.

**Version:**
:   Sun Jan 11 11:41:39 NZDT 2009

---

**Field Summary**

**Fields inherited from class java\_cup.runtime.lr\_parser**

`symbolFactory`


**Constructor Summary**

`Parser()`
  
          Default constructor.

`Parser(java_cup.runtime.Scanner s)`
  
          Constructor which sets the default scanner.

`Parser(java_cup.runtime.Scanner s,
java_cup.runtime.SymbolFactory sf)`
  
          Constructor which sets the default scanner.


| **Method Summary** | |
| --- | --- |
| `short[][]` | `action_table()`             Access to parse-action table. |
| `java_cup.runtime.Symbol` | `do_action(int act_num, java_cup.runtime.lr_parser parser, java.util.Stack stack, int top)`             Invoke a user supplied parse action. |
| `int` | `EOF_sym()`             `EOF` Symbol index. |
| `int` | `error_sym()`             `error` Symbol index. |
| `static Instances` | `filter(java.lang.String expression, Instances input)`             Filters the input dataset against the provided expression. |
| `java.util.Hashtable` | `getAttributeTypes()`             Returns the current attribute - attribute-type relation in use. |
| `java.lang.Boolean` | `getResult()`             Returns the result of the evaluation. |
| `java.util.HashMap` | `getSymbols()`             Returns the current variable - value relation in use. |
| `static java.lang.Object` | `getValue(Instance instance, int index)`             Returns either a String object for nominal attributes or a Double for numeric ones. |
| `static void` | `main(java.lang.String[] args)`             Runs the parser from commandline. |
| `short[][]` | `production_table()`             Access to production table. |
| `short[][]` | `reduce_table()`             Access to `reduce_goto` table. |
| `void` | `setAttributeTypes(java.util.Hashtable value)`             Sets the attribute - attribute-type relation to use. |
| `void` | `setResult(java.lang.Boolean value)`             Sets the result of the evaluation. |
| `void` | `setSymbols(java.util.HashMap value)`             Sets the variable - value relation to use. |
| `int` | `start_production()`             Indicates start production. |
| `int` | `start_state()`             Indicates start state. |

**Methods inherited from class java\_cup.runtime.lr\_parser**

`debug_message, debug_parse, debug_reduce, debug_shift, debug_stack, done_parsing, dump_stack, getScanner, getSymbolFactory, parse, report_error, report_fatal_error, scan, setScanner, syntax_error, unrecovered_syntax_error, user_init`

**Methods inherited from class java.lang.Object**

`equals, getClass, hashCode, notify, notifyAll, toString, wait, wait, wait`

**Constructor Detail**

### Parser

```
public Parser()
```

:   Default constructor.

---


### Parser

```
public Parser(java_cup.runtime.Scanner s)
```

:   Constructor which sets the default scanner.

---


### Parser

```
public Parser(java_cup.runtime.Scanner s,
              java_cup.runtime.SymbolFactory sf)
```

:   Constructor which sets the default scanner.


**Method Detail**

### production\_table

```
public short[][] production_table()
```

:   Access to production table.

    :   **Specified by:**: `production_table` in class `java_cup.runtime.lr_parser`

---


### action\_table

```
public short[][] action_table()
```

:   Access to parse-action table.

    :   **Specified by:**: `action_table` in class `java_cup.runtime.lr_parser`

---


### reduce\_table

```
public short[][] reduce_table()
```

:   Access to `reduce_goto` table.

    :   **Specified by:**: `reduce_table` in class `java_cup.runtime.lr_parser`

---


### do\_action

```
public java_cup.runtime.Symbol do_action(int act_num,
                                         java_cup.runtime.lr_parser parser,
                                         java.util.Stack stack,
                                         int top)
                                  throws java.lang.Exception
```

:   Invoke a user supplied parse action.

    :   **Specified by:**: `do_action` in class `java_cup.runtime.lr_parser`
    :   **Throws:**: `java.lang.Exception`

---


### start\_state

```
public int start_state()
```

:   Indicates start state.

    :   **Specified by:**: `start_state` in class `java_cup.runtime.lr_parser`

---


### start\_production

```
public int start_production()
```

:   Indicates start production.

    :   **Specified by:**: `start_production` in class `java_cup.runtime.lr_parser`

---


### EOF\_sym

```
public int EOF_sym()
```

:   `EOF` Symbol index.

    :   **Specified by:**: `EOF_sym` in class `java_cup.runtime.lr_parser`

---


### error\_sym

```
public int error_sym()
```

:   `error` Symbol index.

    :   **Specified by:**: `error_sym` in class `java_cup.runtime.lr_parser`

---


### setSymbols

```
public void setSymbols(java.util.HashMap value)
```

:   Sets the variable - value relation to use.

    :   **Parameters:**: `value` - the variable-value relation

---


### getSymbols

```
public java.util.HashMap getSymbols()
```

:   Returns the current variable - value relation in use.

    :   **Returns:**: the variable-value relation

---


### setAttributeTypes

```
public void setAttributeTypes(java.util.Hashtable value)
```

:   Sets the attribute - attribute-type relation to use.

    :   **Parameters:**: `value` - the att - att-type relation

---


### getAttributeTypes

```
public java.util.Hashtable getAttributeTypes()
```

:   Returns the current attribute - attribute-type relation in use.

    :   **Returns:**: the att - att-type relation

---


### setResult

```
public void setResult(java.lang.Boolean value)
```

:   Sets the result of the evaluation.

    :   **Parameters:**: `value` - the result

---


### getResult

```
public java.lang.Boolean getResult()
```

:   Returns the result of the evaluation.

    :   **Returns:**: the result

---


### getValue

```
public static java.lang.Object getValue(Instance instance,
                                        int index)
```

:   Returns either a String object for nominal attributes or a Double for numeric
    ones. For all other attribute types this method throws an Exception.
    It also returns a Double object in case of a missing value (for all
    attribute types!).

    :   **Parameters:**: `instance` - the instance to work on: `index` - the index of the attribute to return **Returns:**: the converted value

---


### filter

```
public static Instances filter(java.lang.String expression,
                               Instances input)
                        throws java.lang.Exception
```

:   Filters the input dataset against the provided expression.

    :   **Parameters:**: `expression` - the expression used for filtering: `input` - the input data **Returns:**: the filtered data **Throws:**: `java.lang.Exception` - if parsing fails

---


### main

```
public static void main(java.lang.String[] args)
                 throws java.lang.Exception
```

:   Runs the parser from commandline. Takes the following arguments:

    1. expression
    2. input file
    3. class index (first|last|num), use 0 to ignore
    4. output file

    :   **Parameters:**: `args` - the commandline arguments **Throws:**: `java.lang.Exception` - if something goes wrong


---


|  |  |  |
| --- | --- | --- |
| **Overview** \| **Package** \| **Class** \| **Tree** \| **Deprecated** \| **Index** \| **Help** \| **Weka's home** | |  |
| PREV CLASS   **NEXT CLASS** | **FRAMES**    **NO FRAMES**     **All Classes** |  |
| SUMMARY: NESTED \| FIELD \| CONSTR \| METHOD | DETAIL: FIELD \| CONSTR \| METHOD |  |


---
